# Supplementary figures and images for: Key processes required for the different stages of fungal carnivory by a nematode-trapping fungus
Source: PLoS Biol. 2023 Nov 21;21(11):e3002400. doi: 10.1371/journal.pbio.3002400 (PMC10662756; doi:10.1371/journal.pbio.3002400)

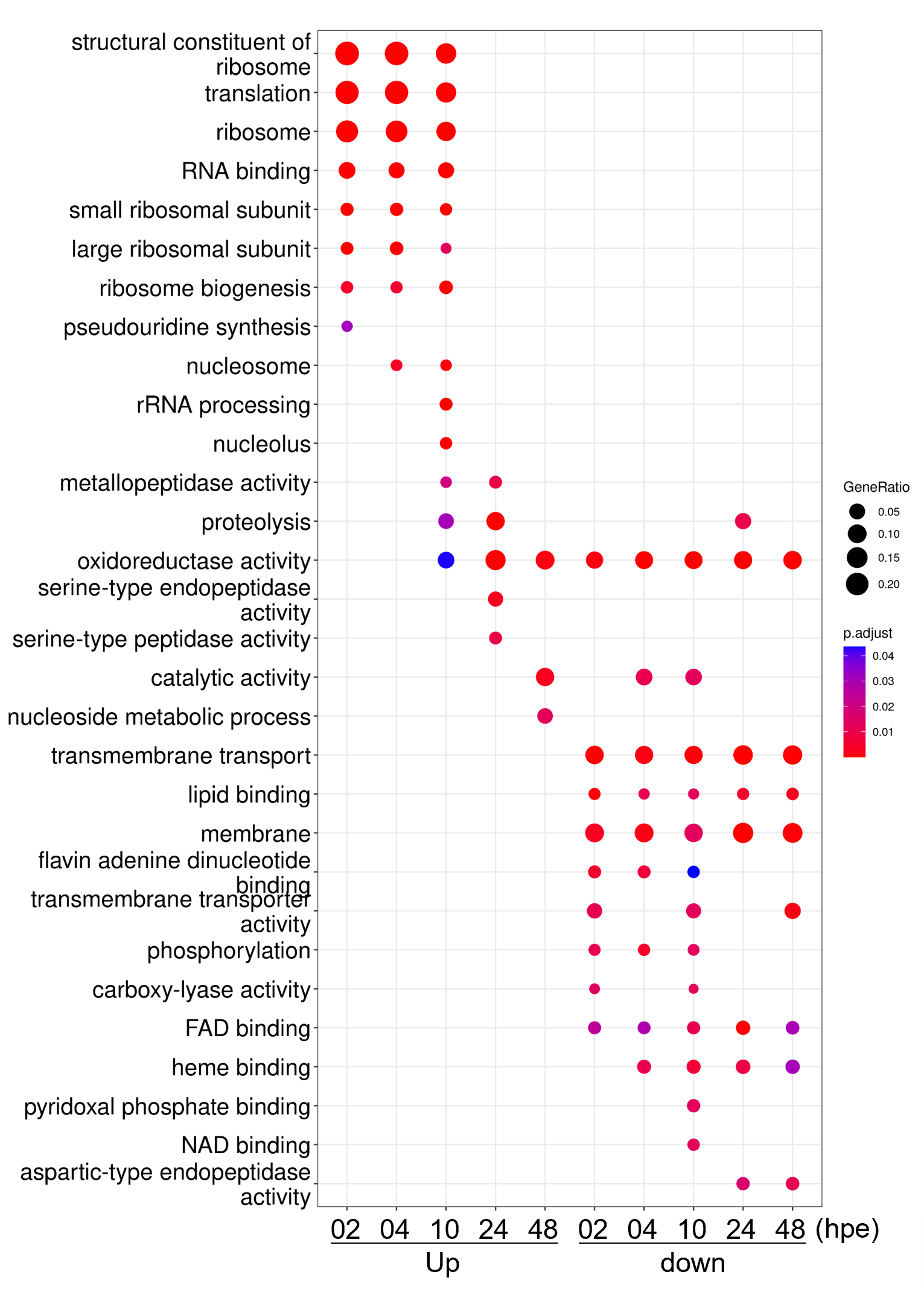

Supplement: S1 Fig — Gene Ontology categories for each time point with a p-value < 0.05. The data underlying this Figure can be found in S1 Data. (TIF) [file pbio.3002400.s007.tif]

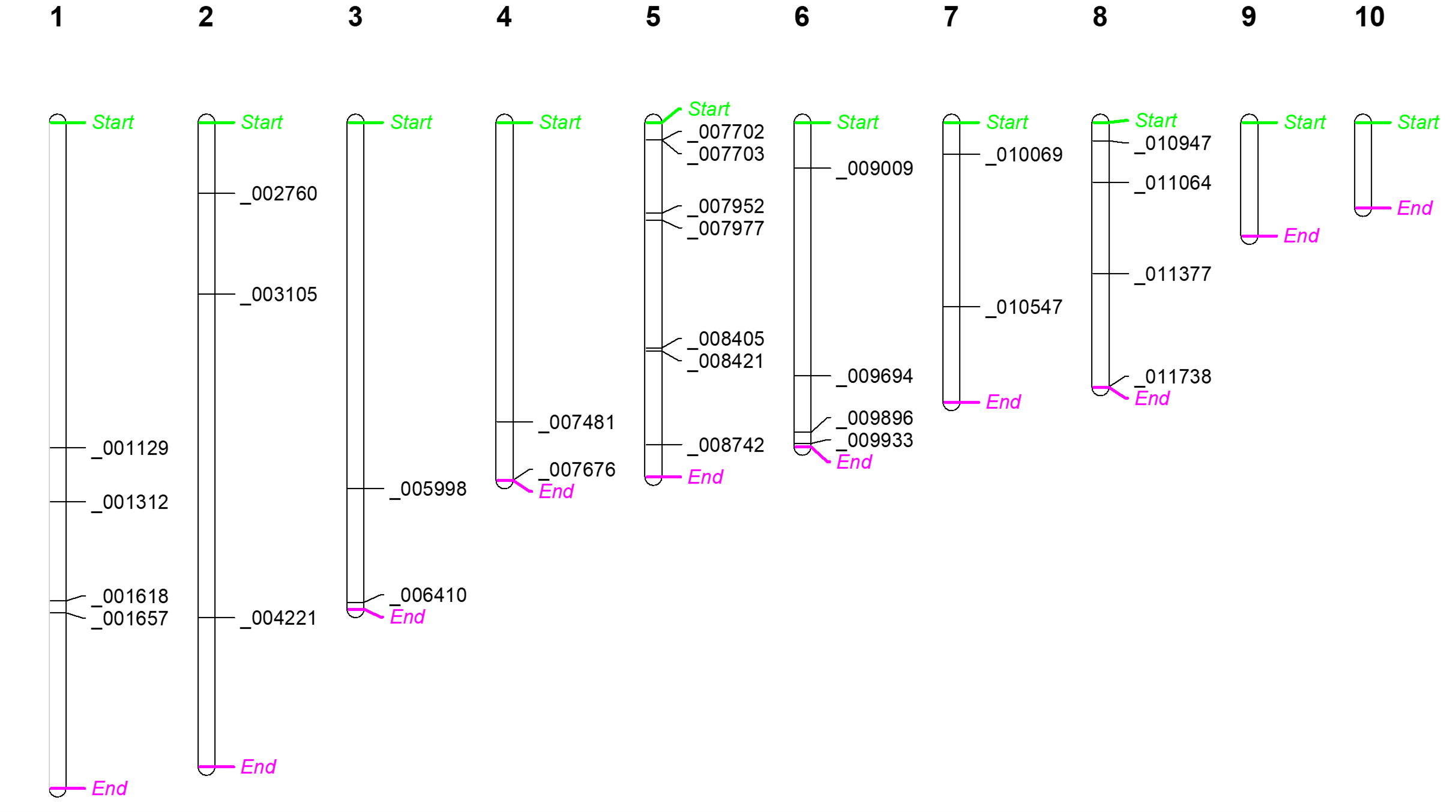

Supplement: S2 Fig — (TIF) [file pbio.3002400.s008.tif]

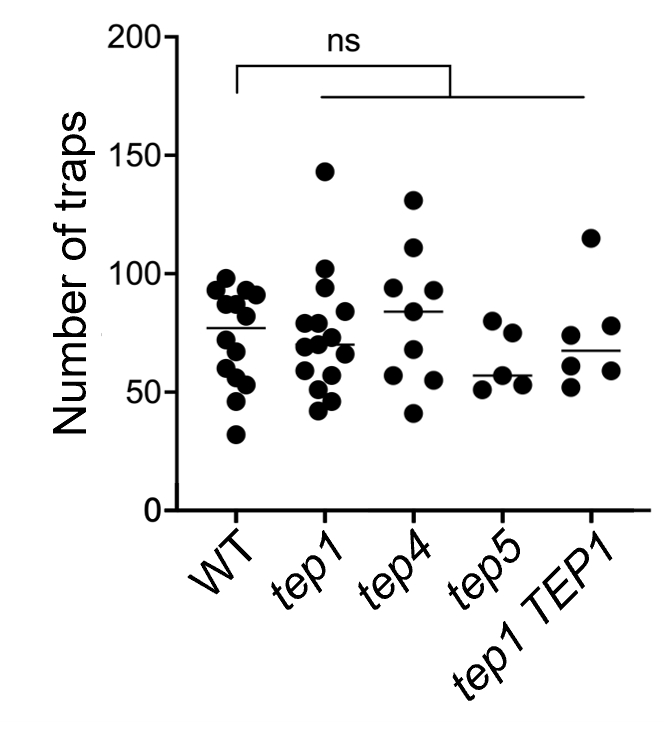

Supplement: S4 Fig — The data underlying this Figure can be found in S1 Data. (TIF) [file pbio.3002400.s010.tif]

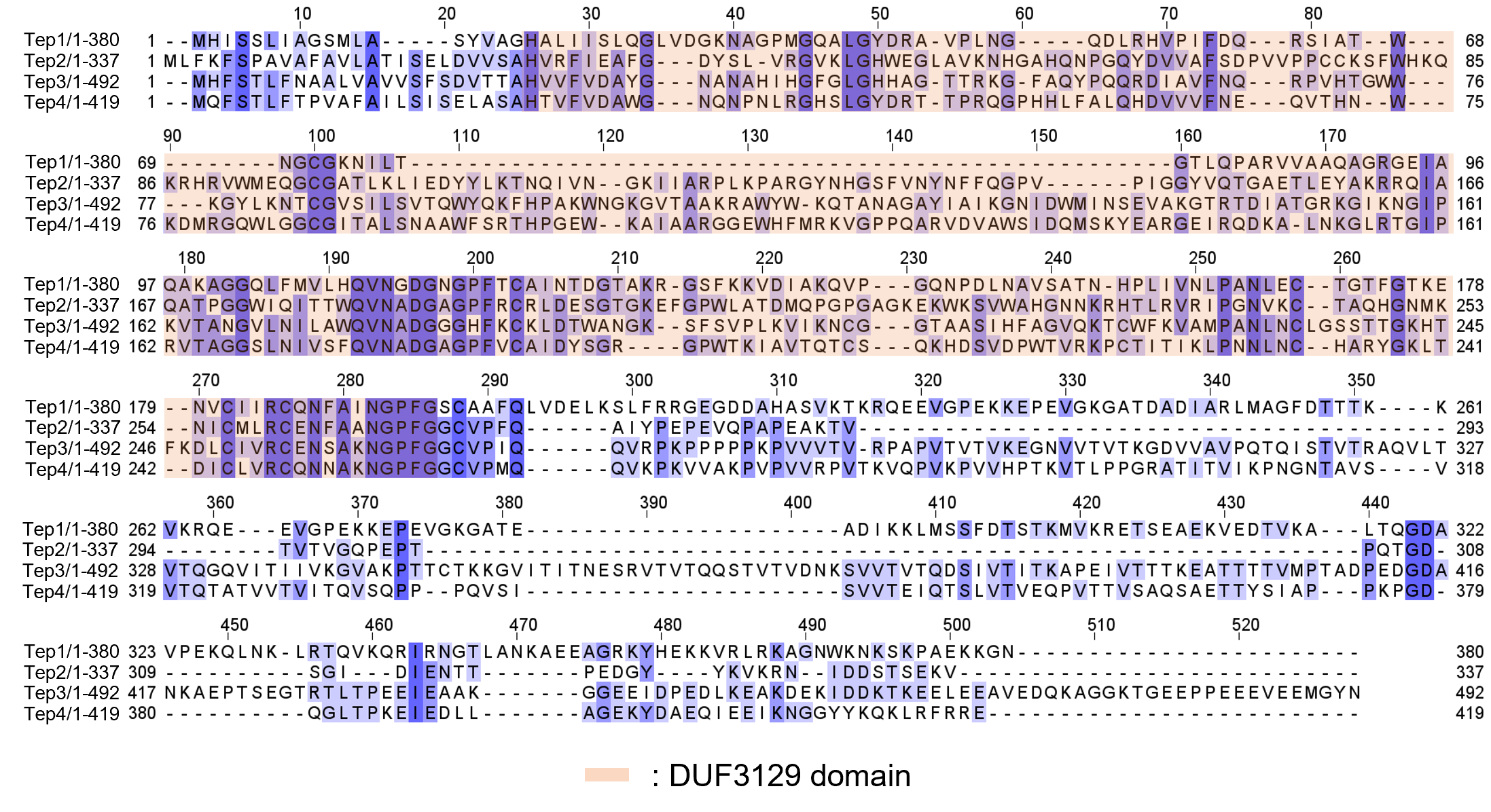

Supplement: S5 Fig — (TIF) [file pbio.3002400.s011.tif]

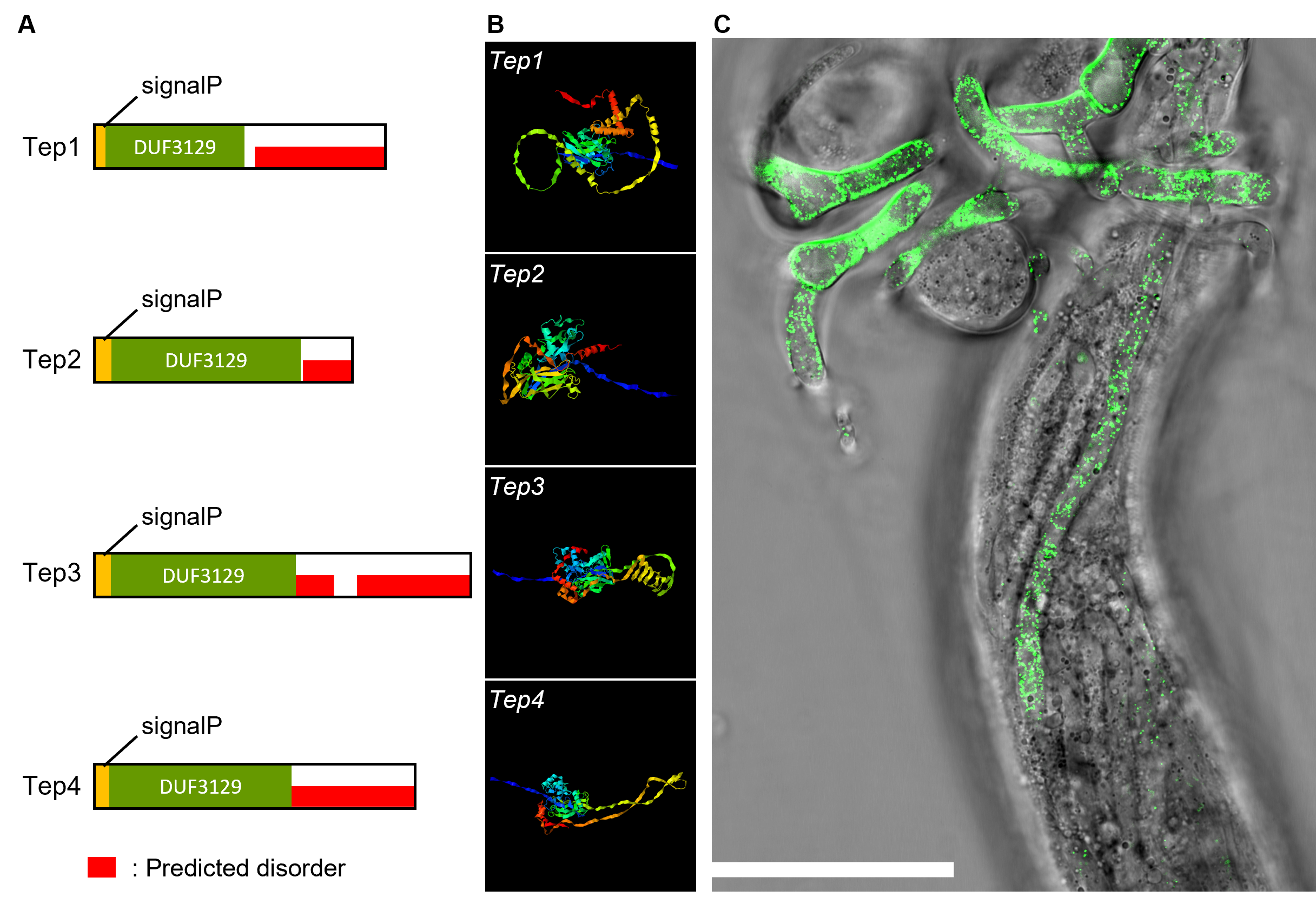

Supplement: S6 Fig — (A, B) Gene model (A) and protein structure prediction (B) of the top 4 expressed TEP proteins of A. oligospora. (C) The confocal image of close-up look of trap induction of the TEP1-GFP strain (scale bar, 50 μm). (TIF) [file pbio.3002400.s012.tif]

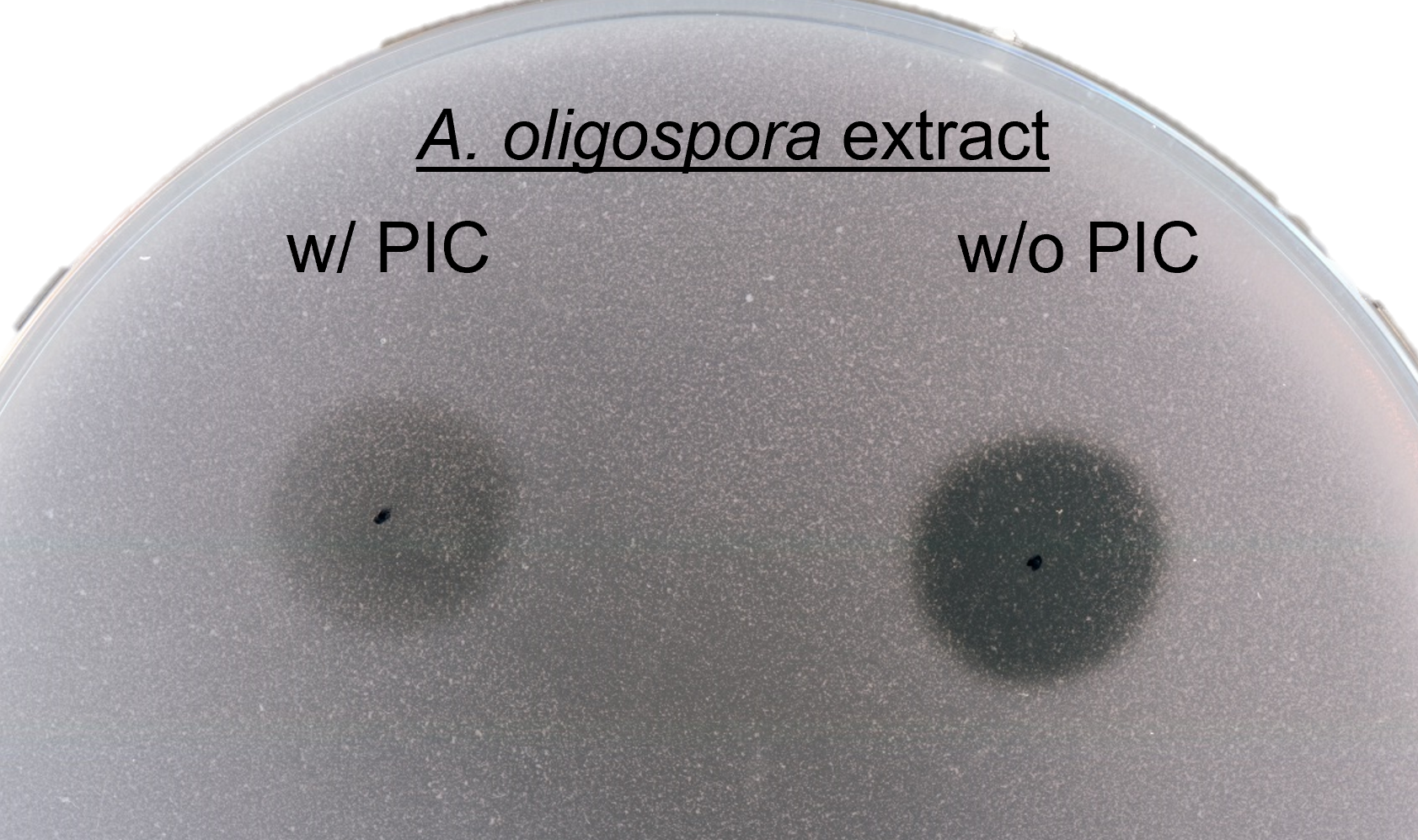

Supplement: S7 Fig — A comparison between samples subjected to PIC treatment (left) and samples without PIC treatment (right), illustrating the presence of clear zones. (TIF) [file pbio.3002400.s013.tif]

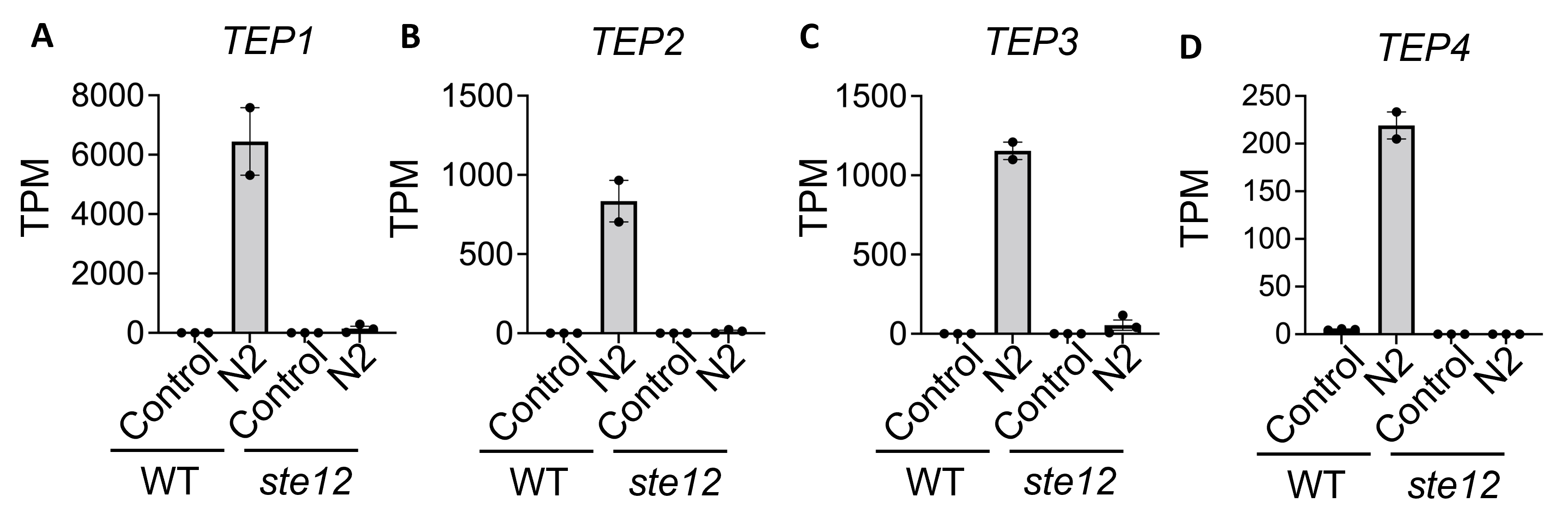

Supplement: S8 Fig — The data underlying this Figure can be found in S1 Data. (TIF) [file pbio.3002400.s014.tif]

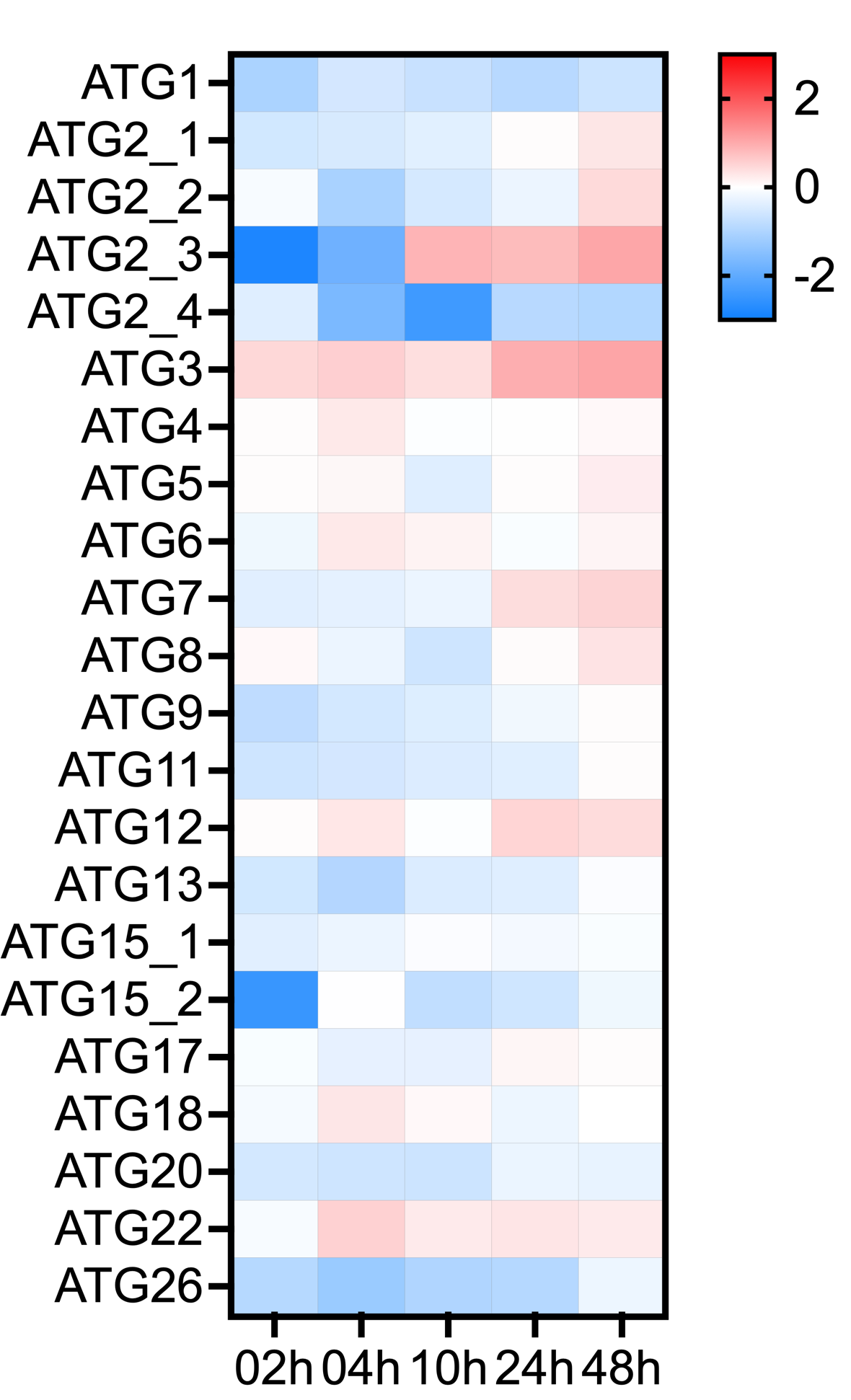

Supplement: S9 Fig — The data underlying this Figure can be found in S1 Data. (TIFF) [file pbio.3002400.s015.tiff]
