# Supplementary material for: Key processes required for the different stages of fungal carnivory by a nematode-trapping fungus
Source: PLoS Biol. 2023 Nov 21;21(11):e3002400. doi: 10.1371/journal.pbio.3002400 (PMC10662756; doi:10.1371/journal.pbio.3002400)
Supplement: Attachment — Submitted filename: rebuttal_20230920.pdf [file pbio.3002400.s020.pdf]

Subject: Rebuttal Letter for Manuscript ID PBIOLOGY-D-23-01368R1

Dear Dr. Roberts,

We appreciate the thoughtful feedback provided by the reviewers and the opportunity to revise and resubmit our work. We have carefully gone over the reviewers' comments and suggestions, and addressed these concerns in the revised manuscript. Below, we provide a point-by-point response to the reviewers' comments:

Reviewer #1:

Evolution of diverse lifestyles is an exciting question. In the Ascomycetes, the nematode trapping fungi exhibit the unique ability to switch their morphology and physiology in order to trap and digest nematode prey. The authors in this manuscript used time course transcriptomics to identify gene sets that are differentially expressed at different stages of the trapping. They identified at least three major groups: ribosomal related genes in the early stage (0-10 hrs), secreted proteins in the intermediate stage (4-24 hrs) and proteases in the late stage (10-48 hrs). This result provided rich information about the biological changes in the fungal cells after exposure to the nematode (and during starvation). They performed extensive follow-up studies to expand and validate their RNA-seq results. Using rapamycin treatment, they validated the importance of protein synthesis for trap development. Using mutants defective in protein secretion, they confirmed the importance of the secretome to trapping the prey. Using protease inhibitor cocktails and following the invasive growth of the fungus, they confirmed the role of proteases in the late stage of the predation. Overall, the study enriched our understanding about this unique trait and may have implications for the evolution of carnivorous behaviors in plants and fungi in general.

My major critique and suggestion focus on the section describing the evolutionary features of the DUF3129 gene family. I find the question of how the DUF3129 domain containing genes evolved and their potential relationship with the evolution of the unique trapping trait fascinating. However, I think the methods and results are not well described for me to be convinced of their conclusions, i.e., a clade-sepcific expansion and rapid diversification of the "family" contributed to the phenotypic evolution. First, the methods description for this section is very brief. Only a reference for the "orthogroup" analysis was provided, with no specifics on how the analysis was performed, the parameters used, and why the tool is appropriate for their question. The authors stated that they searched 20 "complete" genomes in the main text, while in Figure 4, they showed an analysis that involved 485 fungal genomes. I cannot find information on the species and genome assembly for these species. Since orthology mapping

results depend highly on the quality of the assembly and completeness of the genome annotation, I cannot assess whether their conclusion on the uniquely large number of genes containing the DUF domain is reliable. Also, Figure 4B is an orthology map, but didn't reveal the species relationship, making it hard to assess whether the family may have expanded before the NFT clade and lost in other species, or whether it expanded specifically in the former. My suggestions for properly supporting their evolutionary statements are:

1. Carefully choose a set of species that span the Ascomycotetes, with priority given to genomes with comparably high quality of assembly and annotation while considering their life traits relevant for this study (so, ok to sample pathogens, but also need to include related non-pathogens).

We thank the reviewer for this suggestion. In the revised manuscript, we have retrieved 2504 fungal genomes from the JGI genome portal and meticulously selected a comprehensive set of 19 diverse fungal species with high quality genomes for the genomic analyses. The full information about the selected genomes is listed in Table S5 and lines 487-505.

2. Explain in detail how the orthogroup analysis was performed, with potential caveats. For a group of key genomes, say the 20 "complete genomes", provide the thresholds used to call hits and the hit list, along with the genome assembly ID information for independent validation.

Relying on our new set of 19 high-quality fungal genomes, we analyzed the relationship of DUF3129 genes among different fungal species. For this, we clustered all genes from the 19 genomes into orthogroups using funannotate_compare (basically a wrap up of ProteinOrtho v5 with options -synteny -cpus=24 -singles -selfblast). Orthogroups containing at least one DUF3129 gene were selected to generate Fig 4C illustrating the distribution of DUF3129 genes among the selected genomes. To identify DUF3129 domain-containing genes, we used the DUF3129 domain of the TEP1 gene as a query in blastp searches. For the 2504 fungal genomes, this search was conducted directly on the JGI website, while for the 19 high-quality genomes, we performed local searches (blast 2.9.0). In the updated version of the manuscript, we have included the NCBI accession numbers for the 19 selected genomes (S5 Table) along with statistics detailing the completeness of these genome assemblies and expanded the methods section to include the exact programs and parameters used in the DUF3129 domain searches and to compute orthogroups (lines 171-192).

3. Complement the above analysis with BLAST or HMMER searches of all the genomes to assess whether the gene number results can be independently verified.

We agree that further sequence search was necessary to confirm the robustness of our results. In addition to the analysis against 19 fungal genomes, a BLAST search was newly performed on the 2504 fungal genomes in the JGI database. We compared the results

from our local analysis on the high-quality genomes and those of JGI and verified that the number of DUF3129 hits was consistent among them. The only discrepancies came from *Penicillium chrysogenum* (2 DUF genes in JGI versus 1 gene in high-quality genomes analysis) and *A. oligospora* (32 DUF genes in JGI versus 27 in high-quality genomes analysis). The difference in these numbers is due to the strains in JGI being different than those with the best quality genomes in NCBI. However, using our local pipeline on the JGI strains for these species yield the same number of blast hits therefore validating the accuracy of our results. The species and strains used in our analysis, as well as, the exact number of DUF3129 genes per species can be found in S5 Table. In addition, the methods used to compute these numbers is detailed in the updated manuscript lines 171-192.

4. For the "expansion" claim, they need to reconstruct the evolutionary history of this gene family (the definition of the family also needs to be defined, depending on if all DUF3129 containing genes have that domain alone or whether there are more complex domain architectures, which could complicate the evolutionary analysis). Using gene tree and species tree reconciliation, they can then infer the timing of the gene duplications and losses, in order to distinguish between early-duplication-followed-by-loss vs lineage-specific duplication hypotheses.

We thank the reviewer for highlighting the importance of investigating the evolutionary history of the DUF3129 gene family. To elucidate the timing of the gene duplications and losses, we retrieved all DUF3129 containing genes from 19 fungal genomes as gene family and assessed gene duplication events using gene tree and species tree reconciliation. Our analysis indicates significant DUF3129 gene divergence during NTF speciation, especially within Ascomycete NTF clades. Even within the *A. oligospora* branch post-*A. vermicola* speciation, we identified 15 additional DUF3129 gene duplications. In contrast, non-NTF species showed only a few duplications, with limited DUF3129 genes (typically zero to six) in plant, insect, human, and other nematode pathogen species. This highlights the rapid DUF3129 gene expansion, possibly linked to the predatory lifestyle of nematode-trapping fungi. In our revised manuscript, the methods used to study gene gain and loss patterns are described in the methods section lines 184-191. Additionally, a phylogenetic tree illustrating these results is available in the revised version (S3 Fig) and explained in the text lines 496-505.

5. For the "rapid diversification" claim, I'm not sure what the criteria is for calling this rapid. Is this compared to other genes with similar characteristics? Or just a subjective assessment? Figure S2 contained an alignment that showed relatively poorly aligned regions towards the C-terminus. Without knowing the time of divergence between the proteins aligned, or specific tests for selection, I would conclude that the C-terminus regions are less functionally constrained and thus diverged more rapidly. More generally, sequence divergence alone is not necessarily a "surprise". Follow up analyses or experiments to determine the impact of the

variable sequences are needed to properly interpret that finding.

We appreciate the reviewer's inquiry regarding the term "rapid diversification." To clarify, in the context of our study, rapid diversification refers to the occurrence of gene duplication events primarily within species displaying specific phenotypes. This phenomenon is accompanied by substitutions under a high mutation rate, resulting in alterations in homologs among different species and leading to diverse functional characteristics.

The reviewer points out the less conserved pattern in the C-terminus regions, which is an important consideration. To assess whether such events occurred in the DUF3129 gene family, we conducted an analysis of the gain/loss patterns across DUF3129 orthogroups in 19 fungal genomes. Our analysis supports this definition in several ways:

1. **Gene Duplication: As shown in S3 Fig, our analysis indicates a significant increase in the number of DUF3129 domain-containing genes within the *Arthrobotrys* clade, suggesting that gene duplication events were specific to this clade.**
2. **Low Sequence Homology: S5 Fig demonstrates that four highly expressed DUF3129 genes of *A. oligospora* exhibit low sequence homology, implying potential functional diversity.**
3. **Branch Lengths in the Species Tree: Branch lengths of the *Arthrobotrys* clade in the species tree (S3 Fig) are notably shorter than those of other model fungi. This observation is significant, considering the substantial increase in DUF3129 gene copy numbers, and suggests lineage-specific events attributable to rapid diversification.**

While we acknowledge the need for further investigations and experiments to elucidate the functional implications of these sequence variations, the collective evidence suggests a rapid diversification of DUF3129 genes.

My other question has to do with the secretome analysis. First, I wonder how the authors define "secretome". We know that not all proteins with a signal peptide are secreted outside the cell. Cell wall proteins have the SP but also another signal often at the C-terminus of the protein, to allow it to be inserted into the plasma membrane and later linked to the cell wall. Does the author's definition include both types? I think distinguishing those that are secreted outside the cell and those that are possibly retained on the cell wall has important implications for how they likely contribute to the trapping of the nematode. For example, the authors suggested that the TEP proteins could mediate adhesion through phase-phase separation. How this proposed mechanism would work would depend on whether the protein is anchored on the cell wall or secreted to the vicinity of the fungal cells. Performing a GPI-anchor prediction and separately analyze the secretome (if the definition includes both secreted and cell wall proteins) would help clarify and enrich their results. Second, I'm not familiar with the term "effector" in this particular context, and therefore cannot interpret the "effectome" result. I'd like to suggest that the authors define this term and explain how "EffectorP" works (briefly) to predict them.

We acknowledge the reviewer's point regarding the definition of "secretome". In this study, we define the "secretome" as a collection of proteins that are secreted by the organism into its environment. The secretome is predicted by a combination of tools, including SignalP 5.0, Phobius, TMHMM 2.0, WolfPSort, and MitoFates. These tools identify potential secreted proteins, including both classic and non-canonical secretome. This comprehensive approach filters out non-secreted proteins, transmembrane proteins, and mitochondrial proteins, ensuring an accurate and reliable prediction of the secretome of *A. oligospora* (lines 145-163).

Effectors, on the other hand, are defined as secreted proteins used by pathogens to suppress the host's immune system, facilitating infection and proliferation. In nematode-trapping fungi, effectors are proteins that have the potential to manipulate nematode behavior during trapping. To identify these effectors, we employ the "EffectorP" algorithm, specifically "EffectorP-fungi 3.0", which is designed to predict such proteins within fungal kingdom (lines 164-166).

Reviewer #2:

In this study, Lin and colleagues examine the transcriptional response of a nematode-trapping fungus during the stages of predation. One signature was the ribosome and DNA replication, and the authors show that rapamycin inhibits trap formation, but rapamycin is a general growth inhibitor and has pleiotropic effects on the cell. Similarly, hydroxyurea is a general growth inhibitor as DNA replication is needed for cellular replication. The signature for the predicted effectors seems much more specific, and the data and conclusions regarding the Sso2 mutant is well supported.

Additionally, the authors identified multiple secreted proteins, and showed the importance of secreted effectors in trap formation. Excitingly, the authors identified an expanded family of secreted proteins that are induced upon nematode exposure and are enriched in the traps. These proteins are required for trap function and potentially represent a new class of proteins. The gene expression signatures also pointed to a role for secreted proteases, and both chemical protease inhibition and specific deletion of highly induced proteases resulted in decreased nematode digestion.

Overall, this is a clear, well-written paper with beautiful figures. There are a few minor points that would increase confidence in the interpretation of a few results, but this overall is a

beautiful example of how RNAseq can lead to interesting hypothesis that are then followed up with clear and well-controlled mechanistic experiments.

One limitation of this study is that it relies on GO term annotation to find enriched processes in the RNAseq analysis. However, the total GO term annotation available for A. oligospora is very limited. The best annotated genes are going to be those with clear conservation across ascomycota, but this will ignore all of the organism-specific gene functions and all of the organism-specific genes. There may indeed be a statistically significant enrichment for ribosome biogenesis, but this may also be an artefact of the available data. Based on this, and their chemical data, it would be helpful to show that the dose of inhibitors used is not impacting overall growth.
We greatly appreciate reviewer's comments. Knowing the limitations of GO term annotation in this study we have also used alternative methods and databases, including Pfam and InterPro, to strengthen our functional enrichment analyses. Regarding inhibitor's impact on growth, our preliminary observations indicate that *A. oligospora* grow at a slightly slower rate, when cultured on medium supplemented with 4 μg/ml rapamycin. While this initial observation suggests that the inhibitors may have a modest effect on growth, we acknowledge the need for more comprehensive investigations to draw a definitive conclusion.

Reviewer #3:
[identifies himself as Steven D Harrris]

In this manuscript, the authors exploit an Arthrobotrys oligospora-C. elegans pathosystem to provide novel insight into mechanisms that underlie fungal carnivory. Specifically, in response to nutrient limitation, A. oligospora generates adhesive nets that trap nematodes that are then digested. Although there are a growing number of "model systems" utilized to understand the diverse morphologies involved in nematode trapping, the Arthrobotrys system is one of the better developed examples. Here, a well designed and executed global transcriptomic approach provides an unbiased perspective that enables three major findings; 1) in preparation for trap formation, ribosome biogenesis and nuclear division are upregulated, 2) an expanded family of proteins that possess the DUF3129 domain play a key role in trap function, possibly as adhesive factors, and 3) metalloproteases are needed for normal levels of nematode digestion. In addition to these specific results, the study also provides a large compendium of gene expression throughout the trap development process that will serve as a robust resource for future studies. In my view, these novel results are notable because they represent one of the first uses of an unbiased approach to understanding trap formation and function. It is only through the use of

these types of approaches that real progress will occur in understanding clade-specific morphologies relevant to specific fungal lifestyles.

I do have the following concerns that should be addressed by the authors;

Lines 111-114. It would be helpful if the authors quantified the different stages of trap development as shown in the images provided in Fig. 1. For example, across each replicate, how many worms were trapped at the 10h timepoints (all, 50%)?

At the 10-hour timepoint, approximately 30% of the nematodes were captured (Fig 2C).

Line 113. What do the authors mean by "worms were washed", and why only at these time points?

Between 2 and 4 hours, traps are absent, and nematodes can be removed by washing the plates with ddH2O before we collect the fungal hyphae. However, at 10, 24, and 48 hours, majority of the nematodes are already captured and been digested by *A. oligospora*. We have included this information in our methods lines 117-118.

Line 276. When comparing the new reference strain to the older one, the authors use the term "strongly". In my view, this is too vague. Even though this is described in more detail elsewhere, some additional context would be helpful here.

We agree with the reviewer's point. Since it is unnecessary to refer to the trapping ability of the previous strain ATCC24927 in the manuscript, we have removed this information from the manuscript, specifically in line 307.

Line 335. Given that the fungus is ramping up for trap development under starvation conditions by inducing ribosome biogenesis and nuclear division, it seems reasonable to speculate that autophagy might play a role in scavenging from hyphae that do not form traps. Do the authors detect any signs in their gene expression dates or microscopy analysis that would hint at a role for autophagy?

To see if there are signs in gene expression that implies the involvement of autophagy, we identified genes associated with autophagy (ATG) genes in our time-course RNA-seq datasets and examined their expression level. We do not observe significant differential gene expression of these ATG genes in response to nematodes (S9 Fig). While this observation might imply that autophagy might not be involved in nematode predation, further detailed investigation is required in the future to draw a conclusion.

Lines 327 and 339. Although it seems likely that both TOR and HU operated through their known modes of action to inhibit trap formation, this is not in fact shown by the authors. I would encourage them to provide confirmatory evidence that, for example, HU did block nuclear division as expected.

We have examined the number of nuclei in the trap cells under HU treatment, and found

that the average number of the nuclei decreased to 4 per cell, indicating nuclear division was blocked as expected (Fig 2E).

Figs. 2D, 3E, and 4D. In both cases, it would be helpful if arrowheads were used to denote traps.

We've now included arrowheads and arrows in these figures to highlight the traps and hyphae in Figs. 2E, 3E, and 4D.

Lines 371 and 519. Is there any evidence for genomic clustering of predicted effectors as observed in plant pathogenic fungi? Along the same lines, did the authors observe any patterns in the genomic locations of the amplified DUF3129 family members? For example, are they localized near chromosome ends?

We checked the genomic location of the 27 DUF3129 genes in *A. oligospora* and found them scattered in the genome. They did not cluster on the chromosome ends. We have added this information in S2 Fig and described the results in lines 465-467.

Fig. 3E. Even if described elsewhere, it would be helpful for the reader if the authors noted here what SR2200 is.

We added the full name of the dye (SCRI Renaissance 2200) in Fig 3E legend to clarify. In addition, we reported the ability of the SR2200 dye to stain fungal cell walls without arresting growth in lines 233-234.

Lines 385 and 475. Although complemented controls are provided for the tep1 deletion mutants, they are not for the sso2, mep1, and amp1 deletions. While I see the rational behind this, the inclusion of these controls is typically a key confirmatory step.

We acknowledge the importance of having complementary strains for mutant confirmation and it is especially important for mutants that have exhibited a phenotype. We did have several attempts to obtain the sso2 complementary strain. However, we failed to successfully reintroduce the wild-type gene with the drug selection marker into the mutant background. For the two protease inhibitor mutants, since the phenotype was partial delayed in colonization of the nematodes, and expected, we did not feel that it was essential to perform complementation. In *A. oligospora*, transformation remains highly challenging. We have tried our best whenever it's possible, and we hope that the reviewer can understand this limitation.

Fig. 4G. It is really hard to discern much from these images, even when they are magnified. Arrows and arrowheads might help with denoting key features. Also, in the legend (line 890), the phrase "after 24h of 6h exposure" is not clear.

We revised the legend to provide a clearer description of the timing of exposure in lines 482-483. Traps are indicated with arrowheads in Fig. 4G.

Lines 440-444 and Fig. 5. These could conceivably be shifted to supplemental files.

We agree with this suggestion and have moved this figure to the supplemental file S6 Fig. The adapted text can be found in lines 535-541.

Line 463. I would suggest that the authors provide some indication of the effectiveness of the PIC cocktail. The delay in digestion would make sense if the cocktail only partially blocked protease activity. If it caused full inhibition, it would imply that other (perhaps non-proteolytic processes) are involved.

We have conducted additional inhibition assays to evaluate the effectiveness of the PIC and confirmed that the cocktail partially blocked protease activity (S7 Fig and lines 570-573).

Lines 484-489. The authors should be careful with the use of the term ploidy. Their results document local increases in nuclear numbers. They provide no data on whether the nuclei themselves are haploid, diploid, etc. I would simply state nuclear numbers instead of ploidy.

We agree with this suggestion and have revised the manuscript accordingly. Instead of "ploidy," we changed it to the number of nuclei (lines 589-599).

General comment. Do the authors know whether localized nuclear divisions themselves serve as a cue for subsequent trap formation? For example, in the HU experiment, does the presence of traps (albeit last lower levels than untreated controls) indicate that their formation is uncoupled from nuclear division, or is it simply a technical issue (i.e., ensuring that all regions of hyphae experience similar doses of HU)?

We thank the reviewer for this interesting observation. We have not yet investigated the potential uncoupling of trap formation from localized nuclei divisions however we will consider this aspect in our future research to better understand the relationship between these processes.

We believe that the revisions we have made strengthen the manuscript and addressed the reviewers' comments comprehensively. We are confident that our work makes a significant contribution to the field and aligns well with the scope and objectives of PLOS Biology.

Once again, we would like to thank the reviewers and the editorial team for their time and consideration.

Sincerely,
Yen-Ping Hsueh
