# Supplementary material for: Key processes required for the different stages of fungal carnivory by a nematode-trapping fungus
Source: PLoS Biol. 2023 Nov 21;21(11):e3002400. doi: 10.1371/journal.pbio.3002400 (PMC10662756; doi:10.1371/journal.pbio.3002400)
Supplement: Attachment — Submitted filename: rebuttal_20231023.pdf [file pbio.3002400.s021.pdf]

Subject: Rebuttal Letter for Manuscript ID PBIOLOGY-D-23-01368R2

Dear Dr. Roberts,

We would like to express our gratitude to you and the reviewers for their thoughtful evaluation of our manuscript. We are grateful for the constructive feedback provided by Reviewer #1, which has improved the quality and clarity of our manuscript.

In response to the reviewer's comments, we have carefully addressed each of the raised points. Here is a summary of our responses:

Reviewer #1:

I appreciate the authors taking the suggestions seriously and did a thorough revision to address them. I'm overall satisfied with their response and believe that the manuscript is much stronger as a result. In particular, the gene tree and species tree reconciliation analysis revealed a clade specific expansion of the DUF family, significantly enhancing the claim that this family's expansion likely contributed to their unique lifestyle. I have a few further suggestions for the authors to consider:

Proper use of "diverge" and "diversification". In the response to my comments and also in the revised text, the authors seem to use "diverge" and "diversify" not only to refer to sequence divergence, but also to refer to gene duplications and losses. To me, these two terms imply sequence evolution unless a specific explanation is provided beforehand. Thus, their use in the text leads to confusion. For example, on lines 496-497, they stated "these DUF3129 genes diverged significantly during the speciation process within NTF (Fig.S3)", but the evidence in Fig. S3 actually showed gene duplication and loss history, with no information on sequence divergence. I suggest changing the text to "the family of genes containing DUF3129 expanded specifically within the NTF. This association implies that their expansion, followed by sequence divergence, likely contributed to the unique biology of the NTF species.".

We appreciate the reviewer’s suggestion. Consequently, we have adapted the text to avoid the term diversification and focus on gene duplications and losses. Specifically, we adapted the text to "the family of genes containing DUF3129 expanded specifically within the NTF. This association implies that their expansion, followed by sequence divergence, likely contributed to the unique biology of the NTF species." in lines 507-509. As well as, adapting line 511 from "Notably, even within the *A. oligospora* branch, which diverged after the speciation with *A. vermicola*, we detected an additional 15 DUF3129 gene duplication events." to "Notably, even within the *A. oligospora* branch, we detected an additional 15 DUF3129 gene duplication events.

This demonstrates that gene expansion continues to occur after the speciation from *A. vermicola*.".

Another point of clarification: in the reply to my comments on "rapid diversification", the authors listed low sequence homology and short branch lengths in the species tree as evidence. Low sequence homology, especially when it applies to some parts of an alignment, can be explained by lack of functional and hence evolutionary constraint, and thus doesn't necessarily suggest functional diversification. The argument on short branch lengths of the species tree is difficult to follow, and the method for this figure leaves me wondering what the branch lengths actually represent - amount of molecular changes in the DUF family genes, or is the species tree based on concatenation of single-copy orthologroups?

We apologize for the lack of sufficient information. The species tree is based on concatenation of single-copy orthologs. Therefore, the branch lengths reflect sequence differences in the single-copy orthologs between species. We added this information and edited the methods regarding S3 Fig (lines 184-195) to better explain how the tree was constructed. As for the "rapid diversification" claim, we decided to exclude it from the manuscript to avoid misunderstandings.

Otherwise, I found the revision satisfactory and would like to congratulate the authors in making this important contribution!

Reviewer #3:
[identifies himself as Steven D. Harris]

The authors have adequately addressed concerns that were identified in the previous version of this manuscript. In my view, it is now suitable for publication.

In conclusion, we believe that these revisions have significantly improved the quality and transparency of our manuscript, and we're confident it is now suitable for publication.

Thank you for your consideration, and we look forward to seeing our story accepted by PLOS Biology.

Sincerely,
Yen-Ping Hsueh
